# Plasticity of Anterior Pituitary Gonadotrope Cells Facilitates the Pre-Ovulatory LH Surge

**DOI:** 10.3389/fendo.2020.616053

**Published:** 2021-02-04

**Authors:** Colin M. Clay, Brian D. Cherrington, Amy M. Navratil

**Affiliations:** ^1^ Department of Biomedical Science, Colorado State University, Fort Collins, CO, United States; ^2^ Department of Zoology and Physiology, University of Wyoming, Laramie, WY, United States

**Keywords:** gonadotrope, gonadotropin releasing hormone receptor, plasticity, luteinizing hormone, luteinizing hormone surge, fertility

## Abstract

Gonadotropes cells located in the anterior pituitary gland are critical for reproductive fitness. A rapid surge in the serum concentration of luteinizing hormone (LH) secreted by anterior pituitary gonadotropes is essential for stimulating ovulation and is thus required for a successful pregnancy. To meet the requirements to mount the LH surge, gonadotrope cells display plasticity at the cellular, molecular and morphological level. First, gonadotrope cells heighten their sensitivity to an increasing frequency of hypothalamic GnRH pulses by dynamically elevating the expression of the GnRH receptor (GnRHR). Following ligand binding, GnRH initiates highly organized intracellular signaling cascades that ultimately promote the synthesis of LH and the trafficking of LH vesicles to the cell periphery. Lastly, gonadotrope cells display morphological plasticity, where there is directed mobilization of cytoskeletal processes towards vascular elements to facilitate rapid LH secretion into peripheral circulation. This mini review discusses the functional and organizational plasticity in gonadotrope cells including changes in sensitivity to GnRH, composition of the GnRHR signaling platform within the plasma membrane, and changes in cellular morphology. Ultimately, multimodal plasticity changes elicited by gonadotropes are critical for the generation of the LH surge, which is required for ovulation.

## Introduction

The anterior pituitary is the body’s master endocrine gland and is, arguably, the most complex endocrine organ in the body. At maturity, this gland is responsible for the synthesis and secretion of hormones, which control homeostasis, growth, lactation, and reproduction in mammals (1). It is composed of five distinct endocrine cell types which secrete six different hormones: gonadotropes release luteinizing hormone (LH) and follicle-stimulating hormone (FSH); thyrotropes release thyroid-stimulating hormone (TSH); corticotropes release adrenocorticotropic hormone (ACTH); somatotropes release growth hormone (GH); and lactotropes release prolactin (Prl) (2). Collectively, anterior pituitary cells are under hypothalamic control and receive feedback from the periphery to adjust hormone secretion to keep the body in homeostatic balance (3).

In mammals, reproductive competence depends on the coordinated activation of gonadotrope cells in response to pulsatile hypothalamic gonadotropin releasing hormone (GnRH) (4, 5). Following release, GnRH is transported *via* the hypophysial portal vessels to the anterior pituitary where it binds to the GnRH receptor (GnRHR) on gonadotropes (6, 7). Stimulation of the GnRHR activates a complex array of intracellular signaling networks that culminates in the synthesis and secretion of the heterodimeric glycoproteins, LH and FSH, into systemic circulation where they regulate gonadal development and function (5, 8). The gonadal steroid hormones, estrogen, progesterone, and androgen, participate in a feedback loop with the hypothalamus and the anterior pituitary to regulate GnRH and gonadotropin secretion (9–11). Taken together, any substantive physiological response of gonadotrope cells represents a complex integration of multiple hormones and signaling pathways that culminate in the functional plasticity of distinct gene programs and protein expression over the course of the estrous cycle.

In females, a mid-cycle surge of LH is obligatory for ovulation. The “LH surge” is initiated by a rise in estradiol‐17β (E2) production by the ovarian follicles, triggering a switch from negative feedback on the hypothalamus and pituitary gland to transient positive feedback (5, 11). E2 positive feedback is driven by activation of ERα in kisspeptin neurons in the hypothalamus, which stimulate GnRH neurons to increase GnRH pulse frequency (12–14). In addition, rising E2 levels prior to ovulation stimulate GnRHR expression in multiple model systems (15–17). The dynamic increase in GnRHR expression represents an important priming event, which increases the sensitivity of gonadotropes to GnRH in preparation for the LH surge (18–20). During this critical pre-ovulatory window, gonadotrope cells demonstrate both functional and organizational plasticity to induce the LH surge. At the simplest level, gonadotrope functional plasticity is illustrated by the changes in the intracellular stores of LH and FSH and the relative abundance of secretory granules following GnRH stimulation (21). Collectively then, changes in gonadotrope synthesis and secretion of LH are not only dependent on changes in GnRH pulse frequency, but also the number of GnRH receptors available for binding and, consequently, the responsiveness to a given dose of GnRH. Finally, GnRH and, perhaps, E2, initiate dynamic organizational plasticity within the gonadotrope population that results in the development of cellular processes or projections containing LH that extend toward capillary sinusoids (22, 23). In this mini review we will address each of these dynamic changes that underlie gonadotrope plasticity including altered sensitivity to hypothalamic GnRH, the GnRHR signaling platform, and morphological alterations in the gonadotrope network.

## Gonadotrope Functional Plasticity

### The Gonadotrope Population

Gonadotropes are a minority population of cells representing between 5% and 15% of the anterior pituitary gland and are traditionally defined by a specific phenotype that includes expression of 4 unique genes, the common glycoprotein hormone α subunit, the unique LHβ and FSHβ subunits and the GnRHR (24). Gonadotropes are classically viewed as a single distinct cell lineage, however, as unbiased technological methods have advanced, it is increasingly clear that the gonadotrope population has greater complexity and plasticity with cell-type composition and hormonal expression than previously recognized (25). For example, gonadotropes can be classified as either mono or bihormonal based on their expression of LH and/or FSH (26, 27). Gonadotropes can further be classified by their size as “small”, “medium”, and “large” cells whose percentages display distinct changes during different stages of the reproductive cycle (28, 29).

Gonadotrope plasticity across the estrous cycle has been well documented (25, 30). For example, the percentage of bi-hormonal gonadotrope cells dynamically elevates during the pre-ovulatory period when large amounts of gonadotropin are released into systemic circulation (26, 31). Beyond changes in LH and FSH levels in gonadotropes, our current understanding of the pituitary expands beyond mutually exclusive cell lineages to include transdifferentiation. In support of this, somatotrope cells may transition into gonadotropes to support the LH surge by upregulating gonadotropin mRNA expression (32). Gonadotrope population plasticity is also supported by recent single cell RNA-seq analysis which described a unique cluster of cells containing Prl, Gh, Tshb, POMC, and Lhb mRNAs that represents approximately 5%–11% of the total anterior pituitary cell population in mice (33). This result is intriguing as traditionally gonadotropes and corticotropes derive as POU1F1 independent lineages during embryonic development (34). The developmental origins and transdifferentiation mechanisms of these multi-hormonal cell clusters is uncertain but clearly warrants further investigation especially considering mRNA levels do not necessarily correlate with protein expression.

Despite this unknown, it is interesting that the multi-hormonal pituitary cells represent a shift-able pool of cells that alter gene programs depending on the physiological state. For example, Prl expression in the population is significantly upregulated during pregnancy to initiate and sustain lactation postpartum. Concurrently, the multi-hormonal cells downregulate Lhb to impair ovulation and fertility during the lactation state (33). Thus, this multi-hormonal cell population is dynamic and functions to alter endocrine output to meet physiological demand. It is an intriguing possibility that these multi-hormonal cells may play a role in supporting the LH surge required for ovulation as estradiol has emerged as a stimulus for promoting transdifferentiation (35). Previous studies also found that multi-hormonal anterior pituitary cells are not only more abundant in females compared to males, but also display greater plasticity (33, 36, 37). Thus, female specific plasticity may underlie a broader mechanism under which clusters of multi-hormonal cells adjust their output (LH) based on hypothalamic (GnRH) and sex steroid (E2) hormonal input prior to ovulation to collectively maximize LH production necessary for the surge. Taken together, it is clear that the gonadotrope population represents a complex heterogeneous cell network that integrates multiple physiological queues to modulate gonadotropin secretion.

### GnRH Receptor Expression

Ovulation is perhaps the most fundamental event in reproduction in virtually all female vertebrates. It requires a dramatic 20-30-fold surge of LH released by the anterior pituitary into the peripheral circulation to induce the release of the oocyte from the ovary (38). The dynamic change in GnRHR expression has received particular attention as an important mediator of the LH surge, since it functions to increase the sensitivity of gonadotropes to GnRH during the periovulatory period when serum concentrations of progesterone decline as a result of luteolysis and E2 levels rise with the development of the preovulatory follicle (39). The elevation of ovarian E2 leads to a 3–6 fold increase in GnRHR numbers in gonadotrope cells followed temporally by increased hypothalamic GnRHR secretion (20). It is also clear that the heightened expression of GnRHRs in response to E2 is a result of enhanced transcription of the *Gnrhr* gene (39). Although the mechanisms by which E2 stimulates *Gnrhr* gene expression are not clearly defined, it may be partially through a membrane-initiated signal proceeding through a cAMP-dependent mechanism (40).

Certainly, the loss or dysregulation of hypothalamic input to gonadotropes underlies a number of conditions of hypogonadotropic hypogonadism; however, it is becoming increasingly clear that diminished pituitary sensitivity to GnRH may also contribute to irregular ovulatory cycles. Of particular note is the inverse relationship between GnRHR levels and elevated BMI associated with obesity. Work in both humans and mice suggest that gonadotrope cells are regulated by metabolic signals, including the hormone leptin (41–43). More specifically, Gwen Childs research provides a direct link between leptin signaling and GnRHR expression in mice (41, 44). Leptin resistance, which is characteristic of an obese state, results in a loss of GnRHR expression on the plasma membrane that may reflect a post-transcriptional regulatory mechanism (41). Additional work by Nanette Santoro’s group and others highlights that obesity reduces LH pulse amplitude in women without a corresponding change in LH pulse frequency (43, 45, 46). These women present with a relative hypogonadotropic hypogonadal condition that appears to reflect diminished pituitary sensitivity to GnRH. If correct, then changes in the metabolic state may alter the ability of E2 to either elicit enhanced GnRHR expression or to activate the key gene programs that effectively couple GnRHR activation and increased gonadotropin production. Unfortunately, as gonadotropes only reflect approximately 5%–15% of the total population of endocrine cells in the pituitary, it is difficult to probe the unique transcriptional events evoked by E2 in gonadotropes (24). Recently, however, targeted expression of EGFP followed by Fluorescence Activated Cell Sorting (FACS) and RNA-seq allowed for the first characterization of global changes in gene expression in response to E2. This opens new avenues to explore the impact of pathological conditions (e.g. metabolic state) on E2 regulation of gonadotropes in a non-rodent species (sheep) that displays an estrous cycle that closely mimics the menstrual cycle of women (47).

### GnRH Receptor Organization

Following plasma membrane expression, GnRH-occupied GnRHR couples to Gαq/11 leading to stimulation of phospholipase C, elevation of intracellular free calcium and activation of kinase C (PKC) isoforms (48, 49). These early events underlie GnRH activation of multiple mitogen activated protein kinase (MAPK) signaling cascades including extracellular signal regulated kinase (ERK) (50–52). The ERK signaling module has been studied extensively in gonadotropes and is linked to the transcriptional regulation of the *lhb* gene through induction of the immediate early gene Egr-1 (53–55). It is well documented that faster GnRH pulse frequencies generated during the pre-ovulatory period initiate distinct ERK activation patterns that are responsible for dynamic Egr-1 upregulation necessary for LHβ synthesis required for the LH surge (56, 57). *In vivo* work highlights that the lack of ERK signaling in gonadotrope cells results in an acyclic, anovulatory phenotype in female mice due to the loss of Egr-1 and LHβ subunit expression (55). Thus, GnRH induced activation of ERK is absolutely required for expression of LHβ and ovulation underscoring the importance of this signaling pathway within the female reproductive axis.

It is also clear that proper second messenger signaling in gonadotropes is dependent on GnRHR localization to highly compartmentalized microdomains in the plasma membrane (58). Membrane microdomains efficiently and spatially organize the initiation of intracellular signaling by co-localizing membrane receptors and their cognate downstream signaling components in complexes that, upon ligand activation, co-segregate to form transient signaling platforms (59, 60). Within gonadotropes, the GnRHR and downstream signaling intermediates including c-raf kinase, ERK, calmodulin and Gαq are detectable in microdomains in primary cultures of mouse pituitary cells (58). Additionally, the GnRHR co-immunoprecipitated with ERK from microdomain fractions prepared from mouse pituitary cells suggesting that GnRHR and ERK reside in the same microdomain (61). Functionally this is important because following disruption of gonadotrope microdomain organization, GnRHR is no longer capable of initiating ERK activation (58). Taken together, microdomains serve as a spatial platform that allows for precise organization of a multi-protein scaffold that underlies GnRH initiation of ERK activation. What awaits elucidation is the plasticity of the GnRHR signaling proteome. More specifically, how does composition of the GnRHR signaling platform dynamically change over the course of the estrous cycle?

Fluorophore-tagged GnRH receptors have allowed direct observations of the in-membrane behavior of unoccupied, agonist occupied, and antagonist-occupied receptors (62, 63). Fluorescence resonance energy transfer methods demonstrated that agonist but not antagonist leads to self-association of GnRH receptors in the plasma membrane (62). Thus, GnRH (agonist) induced receptor self-association in gonadotropes likely facilitates assembling multiple discrete GnRHR containing membrane microdomains to transduce a GnRH signal that leads to functional ERK activation and subsequent LH synthesis in gonadotropes.

Dynamic assembly of microdomains within the plasma membrane following GnRH activation suggests a role for the cytoskeleton in mediating their aggregation. Consistent with this, many of the molecular components regulating the actin cytoskeleton have been shown to associate with membrane microdomains (64, 65). An emerging concept is microdomains serve as centers for organizing ligand mediated communication between the plasma membrane and the actin cytoskeleton to control nucleation (66). The cytoskeleton has critical importance in gonadotropes as inhibition of actin remodeling effectively blocks GnRH signaling to ERK but also more acute signaling events such as the opening of L-type calcium channels in the plasma membrane (61, 67). Additionally, the disruption of GnRH-induced actin remodeling events blocks LH secretion from primary murine gonadotrope cells (68). Taken together, there appears to be interdependence between actin reorganization, compartmentalized GnRHR signaling to ERK and GnRH-mediated secretory events. Thus, understanding GnRH regulation of actin dynamics has clear implications for both LH biosynthesis and GnRH mediated release of LH into the bloodstream. Both of these pituitary events are critical for generating the large increase in LH secretion necessary for ovulation.

## Gonadotrope Organizational Plasticity

### The *In Vivo* Gonadotrope Network

In gonadotropes, the cytoskeleton plays a critical role in the regulated release of LH following GnRH stimulation (68, 69). Following ligand binding, the actin network reorganizes to allow vesicles to fuse with the plasma membrane to secrete hormone into the extracellular space. Reserve vesicles then move along microtubules and actin to replenish those vesicles that successfully docked with the plasma membrane (70, 71). *In vivo* and *ex vivo* evidence suggests that GnRH-stimulated gonadotropes are also capable of developing processes that extend directionally toward blood vessels during LH secretory episodes (22, 72, 73). Additionally, our group has demonstrated that primary pituitary cells concentrate LHβ into areas of dynamic membrane reorganization after GnRH treatment and, as previously discussed, disruption of the actin cytoskeleton inhibits LH release (68). These observations suggest that GnRHR engagement of the cytoskeleton not only facilitates the exocytosis of LH granules but also organizes these cells into a favorable spatial orientation to achieve a rapid and pronounced increase in circulating LH *in vivo*.

Towards this end, gonadotrope cells are organized in homotypic and heterotypic cellular networks that are often embedded in connective tissue surrounded by rich vascular networks (74–76). Capillary endothelia are fenestrated facilitating transfer of materials between the interstitium and the blood. Depending on the physiological state of the animal, such as estrous cycle stage, gonadotrope cells display considerable surface area apposed to capillary endothelium (73, 77). At the population level, gonadotropes can undergo plasticity changes that allow for coordination with one another for efficient and robust delivery of gonadotropin into the capillary (22, 74, 78). Previous work suggests that steroid hormones can regulate structural plasticity and cell-to cell connectivity of pituitary cells. For example, following castration, somatotropes within a living pituitary slice were found more clustered and responsive to growth hormone within hours (79). In gonadotropes, pretreatment of *ex vivo* murine pituitary slices with 10 nm E2 was capable of significantly enhancing gonadotrope responsiveness to GnRH with respect to cellular process generation and extension (22). The stimulatory E2 effect on gonadotropes was evident in experiments with long-term (14 h) but not short-term (1.5 h) exposure (22). The time dependence is consistent with upregulation of E2 mediated increases in GnRHR synthesis and increased responsiveness to GnRH necessary to generate the LH surge (80). The timing is also consistent with E2 upregulation of a number of genes involved in cell movement and the cytoskeleton (47).

Lastly, if gonadotropes position themselves to maximize their secretory surface to capillary sinusoids then LH secretory granules would need to be polarized to the side of the gonadotrope nearest to a vascular sinusoid during the preovulatory period. Importantly, this appears to be the case. In ovine pituitary cells, LH granules were polarized in about 20-30% of gonadotropes with this number increasing to 80% during the preovulatory LH surge (77, 81). Additionally, positive feedback from E2 can directly affect the mobilization of LH secretory granules (82). The mechanisms by which GnRH and E2 contribute to LH polarization are unclear but gonadotropes appear “primed” organizationally to support the LH surge. Collectively gonadotrope priming reflects multiple events that include enhanced GnRH responsiveness, transport and polarization of LH granules and increased apposition to the vascular system.

## Conclusions

**Figure 1:**
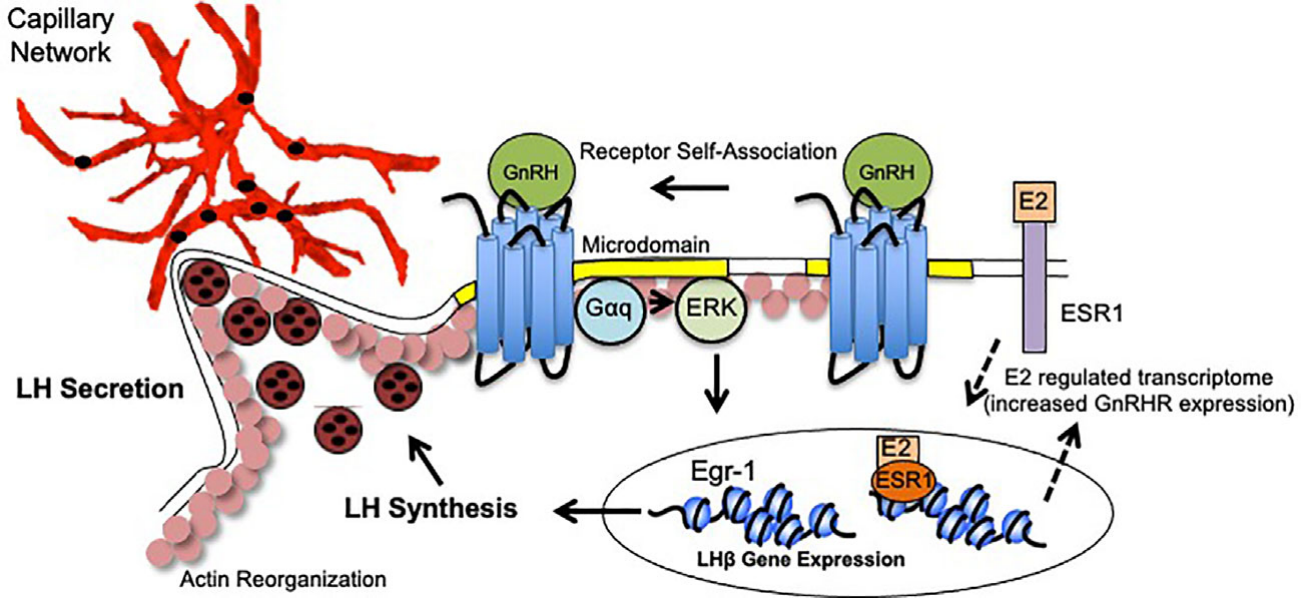
Model of Gonadotrope Dynamics Underlying the LH Surge. During the periovulatory period, ovarian estradiol-17(E2) levels rise. E2 activation of both plasma membrane associated and nuclear ESRI elicits expression of a unique gene program that includes enhanced expression of the GnRH Receptor (GnRHR), and therefore heightened sensitivity to hypothalamic GnRH (dashed arrows). Once expressed, the GnRHR is constitutively localized to microdomains in the plasma membrane (yellow). Following GnRH binding, the GnRHR self-associates to form dimers/oligomers. Second messengers including Gaq/1I and ERK are activated within the microdomains. Increased ERK activity leads to the activation of the immediate early gene Egr-1 which upregulates *lhb* gene expression. Functional LH is synthesized and packaged into secretory vesicles that embed in the cortical actin cytoskeleton (pink circles). GnRH activation also causes reorganization of the actin cytoskeleton which allows for the gonadotropes to appose the capillary endothelium (red) to release LH into circulation (black circles).

The dynamic changes exhibited by gonadotropes in preparation for the LH surge reflects a culmination of events following GnRH binding. These events include enhanced GnRHR responsiveness (driven by elevated E2), GnRHR aggregation, activation of ERK and upregulation of the immediate early gene Egr-1, synthesis and polarization of LH granules, engagement of the actin cytoskeleton, and increased apposition to pituitary vasculature. GnRH, in combination with E2, facilitates plasticity changes that not only underlie LH synthesis, but also to place the gonadotrope population containing LH secretory granules in close proximity to vasculature to maximize circulating LH concentrations. Thus, it is the collective functional and organizational plasticity induced by gonadotropes that mediate the preovulatory LH surge (**Figure 1**).

The mechanisms that underlie the surge in LH release from anterior pituitary gonadotropes have fascinated endocrinologists for decades. It is clear that GnRH and E2 are critical regulators of the LH surge, but mechanistic questions remain regarding how these and other hormones induce plasticity at the cellular, molecular, and gonadotrope network level. For example, how the gonadotrope network communicates *in vivo* to initiate directed movement towards vasculature is still unknown as is the potential role of pituitary cell transdifferentiation during the LH surge. Questions also remain about the role of other steroid regulators, such as progesterone (P4), in inducing the LH surge. Progesterone has historically been thought to suppress gonadotrope function and LH release, although some studies suggest that low P4 levels late in the follicular phase signal the hypothalamus to increase GnRH secretion (83). How this potential mechanism relates directly to gonadotropes requires further studies. Understanding the mechanism that control gonadotrope plasticity is not only of interest at a basic scientific level but also has profound clinical implications from the development of novel birth control and fertility treatments. Future studies will be critical to elucidate the combinatorial effects of endocrine, paracrine, autocrine, and metabolic stimuli on gonadotrope plasticity.

## Author Contributions

All authors contributed to the article and approved the submitted version.

## Funding

This publication was funded by National Institutes of Health, P20GM103432 and R21HD090541 (BC, AN) and R01HD065943 (CC).

## Conflict of Interest

The authors declare that the research was conducted in the absence of any commercial or financial relationships that could be construed as a potential conflict of interest.

## Abbreviations

GnRH, gonadotropin releasing hormone; GnRHR, gonadotropin releasing hormone receptor; LH, luteinizing hormone; FSH, follicle stimulating hormone; MAPK, mitogen activated protein kinase; ERK, extracellular signal regulated kinase.
